# Relevancy Prediction of the Emerging Pathogens with Porcine Diarrhea by Logistic Regression Model

**DOI:** 10.3390/microorganisms13030528

**Published:** 2025-02-27

**Authors:** Benqiang Li, Jie Tao, Xin Li, Jinghua Cheng, Ying Shi, Pan Tang, Huili Liu

**Affiliations:** 1 Institute of Animal Husbandry and Veterinary Medicine, Shanghai Academy of Agricultural Sciences, Shanghai 201106, China; libenqiang2007@163.com (B.L.); livia_taojie@126.com (J.T.); lixinxinli_sadu@outlook.com (X.L.); zero5cheng@163.com (J.C.); shiying@saas.sh.cn (Y.S.); tangpan8811@163.com (P.T.); 2 Shanghai Key Laboratory of Agricultural Genetic Breeding, Shanghai 201106, China; 3 Shanghai Engineering Research Center of Pig Breeding, Shanghai 201302, China

**Keywords:** porcine diarrheal pathogens, mixed infection, emerging pathogens, logistic regression analysis

## Abstract

Porcine viral diarrhea has always been one of the main obstacles to the healthy development of the pig industry in China with its variety of pathogens and complexity of co-infections. Analysis of the dominant mixed-infection model is a fundamental step in boosting the prevention and control of porcine diarrhea. In this study, 3256 porcine fecal samples were collected from 17 pig herds in Shanghai, China, from 2015 to 2023 to identify novel pathogenic infection patterns. The results confirmed that porcine astrovirus (PAstV), porcine sapelovirus (PSV), and porcine epidemic diarrhea virus (PEDV) were the top three agents with positive rates of 28.47%, 20.71%, and 20.23%, respectively. Porcine rotavirus (PoRV) and transmissible gastroenteritis virus (TGEV) accounted for only 8.12% and 1.12%, respectively. Importantly, mixed infection rates were high and complicated. The double infection rate was higher than that of a single infection. Next, the mixed-infection model of PEDV and emerging diarrheal pathogens was explored. The predominant dual-infection models were PEDV/PKoV (porcine kobuvirus) (14.18%), PEDV/PAstV (10.02%), and PEDV/PSV (9.29%). The predominant triple infection models were PEDV/PKoV/PAstV (18.93%), PEDV/PSV/PAstV (10.65%), and PEDV/PKoV/PSV (7.10%). The dominant quadruple-infection model was PEDV/PAstV/PSV/PKoV (46.82%). In conclusion, PEDV is mainly mix-infected with PAstV, PSV, and PKoV in clinical settings. Furthermore, multiple-factor logistic regression analysis confirmed that PAstV, PKoV, bovine viral diarrhea virus (BVDV), and PEDV were closely related to porcine diarrhea. PEDV/PKoV, PEDV/porcine sapovirus (PoSaV), PKoV/BVDV, PoSaV/BVDV, and porcine deltacoronavirus (PDCoV)/PoSaV had great co-infection dominance, which will be helpful for porcine co-infection research.

## 1. Introduction

With the globalization of the pig industry, porcine pathogens are emerging more frequently and are spreading worldwide. Enteric diseases that present with severe diarrhea are the predominant cause of morbidity and mortality in piglets [1]. Although several measures have been taken to prevent swine diarrhea, it remains the most common health problem in the pig industry because of the complexity and unrestrained pathogenic factors [2]. Most of the viruses causing diarrhea outbreaks in neonatal piglets are RNA viruses, including coronaviruses, rotaviruses, picornaviruses, astroviruses, and pestiviruses [3]. As a most important diarrheal pathogen, porcine epidemic diarrhea virus (PEDV) was first identified in China in the 1980s, and, in October 2010, a large-scale PED outbreak caused by a variant of PEDV occurred in China, with a significant increase in morbidity and mortality [4]. In Europe, only classical PEDV was reported sporadically until, in 2014, outbreaks of PEDV were described [5]. This indicates that the damage caused by PEDV has spread worldwide. This was confirmed by metagenome sequencing, which revealed that PEDV viral content in the diarrheal feces of piglets was more than 50%, and the proportion of other coronaviruses was approximately 3% [6]. However, by 2024, members of the *Picornaviridae* family accounted for the majority of the viral communities in piglet diarrheal feces, whereas the presence of coronaviruses was less than 1.7% [7]. This was also consistent with the metagenome sequence data of piglet diarrhea stools collected in our laboratory in 2023 [8].

It has been speculated that the infection pattern of porcine diarrheal pathogens is changing. Reports have shown that the status of PEDV as a major pathogen is decreasing, while the positive rates of emerging diarrheal pathogens are increasing. However, commercially available vaccines are mainly PEDV single vaccines and PEDV/transmissible gastroenteritis virus (TGEV)/porcine rotavirus (PoRV) or PEDV/TGEV polyvalent vaccines, which are not suitable or fully protective against current pathogen infection patterns. This may be a key reason for the unsatisfactory prevention and control of diarrheal diseases in swine. Therefore, the identification of pathogenic infection patterns in swine diarrhea is currently an important area of research.

Logistic regression is a type of multiple regression method used to analyze the relationship between a binary or categorical outcome and multiple influencing factors [9]. In veterinary epidemiology, logistic regression analysis has been used in numerous research investigations involving the study of risk factors, including lambs, goats, minks, poultry, cows, sows, and pigs [10,11,12]. In this study, logistic regression analysis was used to explore the relationship between emerging pathogens and porcine diarrheal disease. This study will provide scientific data for the prevention and control of emerging porcine diarrheal pathogens.

## 2. Materials and Methods

### 2.1. Samples and Multiplex PCR Assay

In this study, 3256 diarrheal stools of the suckling piglets within 28-day-old were collected from seventeen pig herds (named A–Q) in Shanghai, China, between 2015 and 2023. All samples were diluted with PBS and stored at −80 °C in sterile plastic tubes until detection.

According to the manufacturer’s instructions, total RNA of the samples was extracted using TIANamp virus RNA Kit (Tiangen Biotech, Beijing, China) followed by transcription using the PrimeScript Double Strand cDNA Synthesis Kit (Takara, Dalian, China). Then, the cDNA was subjected to multiplex PCR assay to detect 11 porcine diarrhea pathogens, including porcine epidemic diarrhea virus (PEDV), transmissible gastroenteritis virus (TGEV), porcine rotavirus (PoRV), bovine viral diarrhea virus (BVDV), classical swine fever virus (CSFV), porcine sapelovirus (PSV), porcine astrovirus (PAstV), porcine sapovirus (PoSaV), porcine kobuvirus (PKoV), porcine teschovirus (PTV), and porcine torovirus (PToV) [13]. A total of 2 µL cDNA, 25 µL 2× PCR Mix, 0.5 µL each primers (total of eleven pairs of primers, listed in Table 1), and 12 µL H_2_O were mixed in a PCR tube with the amplification program: 95 °C for 5 min; 95 °C for 30 s, 55 °C for 30 s, and 72 °C for 30 s for 25 cycles; 72 °C 5 min. All assays were performed using positive and negative controls. Finally, the detection results were analyzed comprehensively.

### 2.2. Statistical Analysis

The infection status of porcine diarrheal pathogens was defined for each sample as a binary outcome. Tabular methods were used to calculate and map the correlation between emerging diarrheal pathogens and piglet diarrhea using R packages maps [14]. Furthermore, their correlation with coinfection was statistically analyzed. Statistical significance was set at *p* < 0.05. 

## 3. Results

### 3.1. Comparative Analysis of Diarrheal Pathogen Prevalence in Different Pig Farms

**Figure 1:**
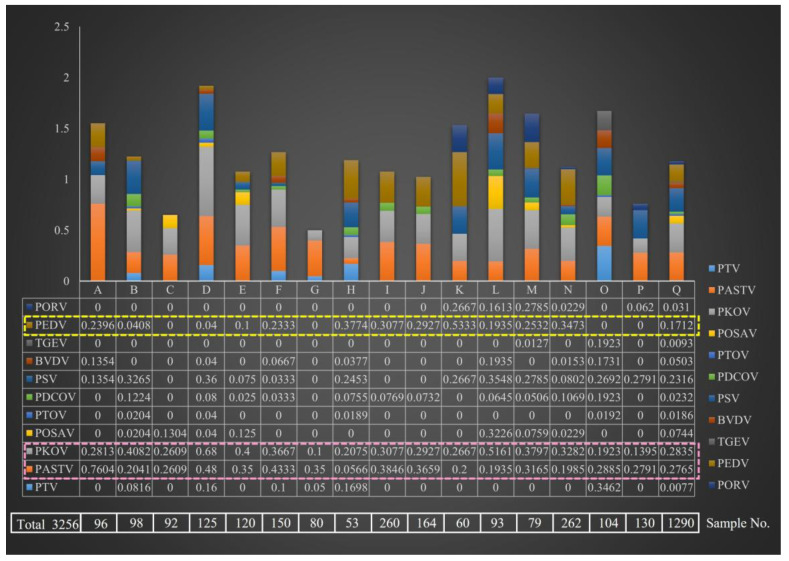
Epidemiological survey of porcine viral diarrheal viruses on different pig farms.

In total, 3256 diarrhea stools were collected from 17 pig farms in Shanghai, China. The multiplex PCR assay showed that the prevalence of diarrheal pathogen was varied among the A–Q farms (Figure 1). The complexity of pathogen composition was Q > D > B = H = L = M = N = O > F > E > A = K > I = J = P > C = G. The Q farm was an important object for monitoring porcine diarrhea disease in which 1290 diarrhea stools were collected. The results showed that all 11 pathogens were detected in farm Q, including classical diarrheal pathogens (PEDV, TGEV, and PoRV) and emerging diarrheal pathogens.

PEDV, an important diarrheal pathogen, was detected in 13 farms, with the highest positive rate (53.33%) in K farm. There were four farms (C, G, O, and P) without PEDV infection (yellow dotted box in Figure 1). However, it is noteworthy that emerging pathogens of PKoV and PAstV were prevalent in all 17 pig farms (pink dotted box in Figure 1). The PKoV-positive rate of farm D (68%) was the highest, and that of farm A (76.04%) was the highest. In general, the prevalence rate of PEDV was 4–53.33%, whereas that of PKoV and PAstV was 10–68% and 5.66~76.04%, respectively. It is speculated that PAstV and PKoV may have been the dominant strains in pig herds, with general upward trends.

### 3.2. Overall Epidemic Trend of Porcine Diarrheal Pathogens in Shanghai from 2015 to 2023

**Figure 2:**
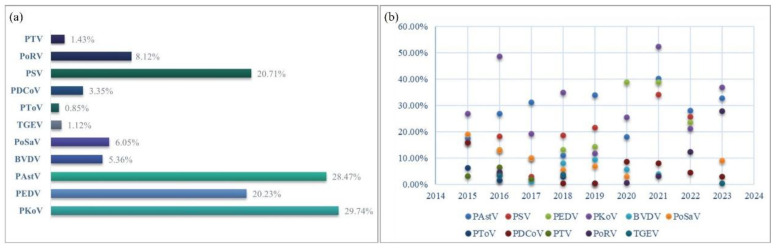
Epidemiological survey of porcine viral diarrheal viruses between 2015 and 2023. The total positive rate (**a**) and annual positive rate (**b**) of the 11 porcine viral diarrheal pathogens were determined.

Based on an early investigation, 11 diarrhea pathogens, including PEDV, TGEV, PoRV, BVDV, CSFV, PSV, PAstV, PoSaV, PKoV, PTV, and PToV, were selected for detecting piglet fecal samples. Continuous pathogen monitoring from 2015 to 2023 revealed that PKoV had the highest positivity rate (29.74%), followed by PAstV (28.47%), PSV (20.71%), and PEDV (20.23%) (Figure 2a). In previous years, PEDV, TGEV, and PoRV were the three most important pathogens causing porcine diarrhea. However, their detection rates have decreased. Compared to the low detection rate of TGEV (1.12%), PoRV had a higher rate of 8.12%. Since its discovery in 2012, PDCoV has been prevalent for several years and was once considered to have the same important status as PEDV. However, the total positive rate was only 3.35%, which was even lower than the BVDV (5.36%) and PoSaV (6.05%) detection rates.

Annual trend analysis showed that at least six types of porcine diarrheal pathogens were present in clinical samples, and up to nine pathogens were detected in 2018 (Figure 2b). It can be clearly seen that PKoV (light purple ball) and PAstV (light blue ball) were the two most dominant pathogens in piglet diarrhea samples with high positive rates in each year (Figure 2b). PSV and PEDV have similar infection advantages, ranking third or fourth, respectively.

### 3.3. Analysis of the Mixed Infection in Porcine Diarrhea Samples

**Figure 3:**
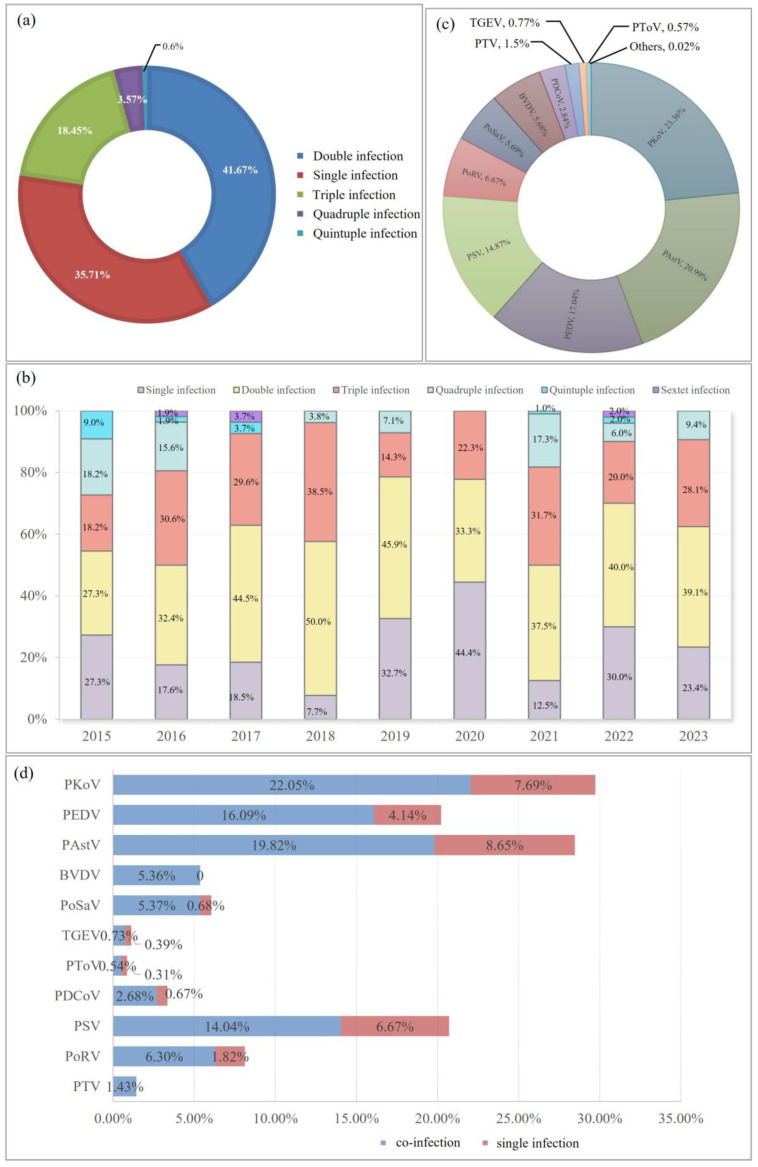
Co-infection survey with porcine viral diarrhea virus. (**a**) Proportion of each infection mode. (**b**) Analysis of infection modes between 2015 and 2023. (**c**) Identification of dominant pathogens in the co-infected samples. (**d**) Comparison between the proportion of single infections and coinfections for each pathogen detected.

Subsequently, infection models of porcine diarrheal pathogens were explored. The proportions of single, double, triple, quadruple, and quintuple infections were 35.71, 41.67, 18.45, 3.57, and 0.6%, respectively (Figure 3a). Double infections have a higher rate than single infections each year from 2015 to 2023, except for 2020 (Figure 3b). In 2018, double and triple infection rates reached 50% and 38.5%, respectively, which were the highest percentage in nine years. This was the only time that a single infection (44.4%) was higher than the double infection rate in 2020 (Figure 3b). This may be related to the COVID-19 pandemic. Improved quarantine measures on farms, and stricter controls on personnel, vehicles, and logistics may result in relatively few outbreaks of pathogens.

Among these mixed infection agents, PKoV (23.36%), PAstV (20.99%), PEDV (17.04%), and PSV (14.87%) were the top four pathogens identified at high proportions in the co-infected samples (Figure 3c).

Analysis from the perspective of a single pathogen showed that the number of mixed-infection samples was 3.88 times that of single-infection samples in PEDV-positive samples (Figure 3d). Except BVDV and PTV with no single infection, the data of PoSaV were biggest (7.88), followed by PDCoV (4) and PoRV (3.46).

### 3.4. Identification of Mixed-Infection Models of PEDV and Emerging Diarrhea Pathogens

**Figure 4:**
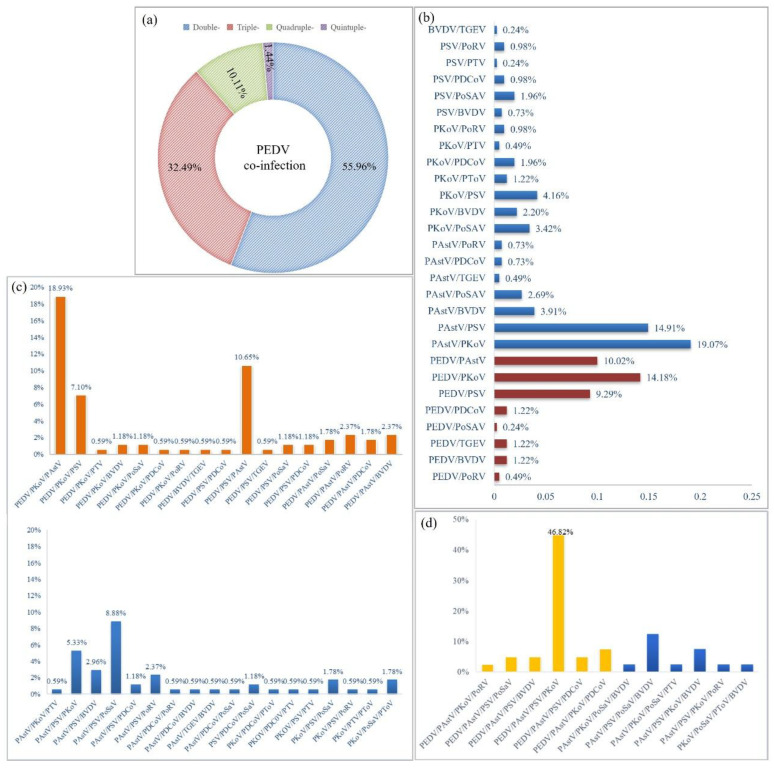
Analysis of multiple-infection patterns of PEDV in porcine diarrhea pathogen infection models. (**a**) Proportion of single, double, triple, and quadruple coinfections. (**b**) Percentage of specific PEDV double coinfections. (**c**) Percentage of specific triple PEDV co-infections. (**d**) Percentages of specific PEDV quadruple co-infections. The orange, red, and yellow columns represent the double-, triple-, and quadruple PEDV infection models and other porcine diarrhea viruses, respectively.

PEDV is a classic and important diarrheal agent involved in porcine diarrheal disease; however, it is always mixed with other diarrheal pathogens. Therefore, mixed infection models of PEDV and emerging diarrheal pathogens were identified. The results confirmed that the PEDV-based double, triple, quadruple, and quintuple infection rates were 55.96, 32.49, 10.11, and 1.44%, respectively (Figure 4a). Over half of the PEDV-positive samples were double-infected (55.96%). In addition, double and triple infections were dominant in PEDV-positive samples, with rates of 32.49% and 10.11%, respectively.

Furthermore, we statistically analyzed all the co-infection models. In double infections, PAstV/PKoV (19.07%) and PAstV/PSV(14.91%) had the highest rates. PEDV/PKoV (14.18%), PEDV/PAstV (10.02%), and PEDV/PSV (9.29%) were the three dominant double-infection models of PEDV (Figure 4b). In triple infection, PEDV/PKoV/PAstV had the highest proportion (18.93%), followed by PEDV/PSV/PAstV (10.65%) and PAstV/PSV/PoSaV (8.88%), while PEDV/PKoV/PSV also presented a relatively high proportion of 7.10% (Figure 4c). PEDV/PKoV/PAstV, PEDV/PSV/PAstV, and PEDV/PKoV/PSV are the predominant triple infection models for PEDV. In quadruple infections, PEDV/PAstV/PSV/PKoV was the only dominant infection model (46.82%) (Figure 4d). In conclusion, PEDV is mainly co-infected with PAstV, PKoV, and PSV in clinical diarrhea samples, which may be the dominant infection model in pig herds.

### 3.5. Logistic Regression Analysis of the Porcine Diarrhea Pathogens

**Figure 5:**
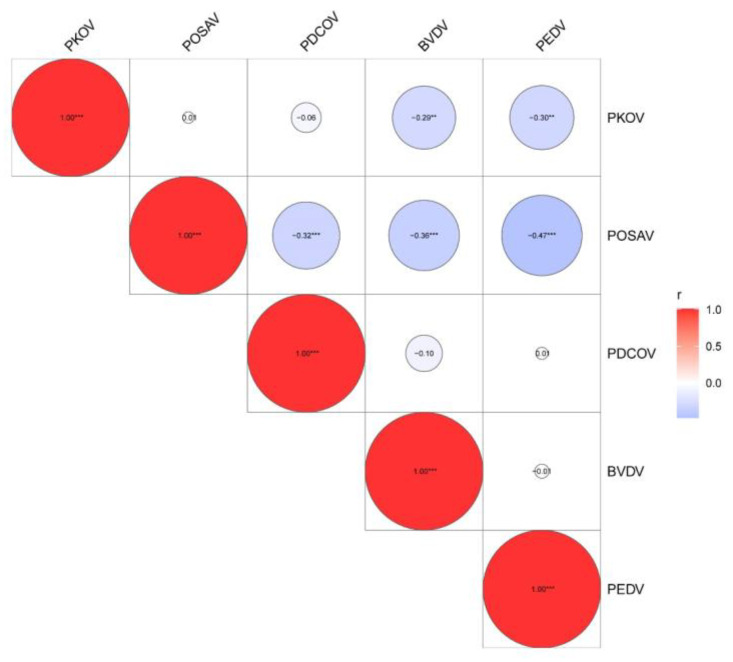
Statistical analysis of the correlation of pathogenic co-infection in swine diarrhea. “**” *p* < 0.01, ” ***” *p* < 0.001.

To explore the correlation between these pathogens and diarrhea, both single-factor and multiple-factor logistic regression analyses were performed. Single-factor regression analysis showed that all 11 pathogens were associated with porcine diarrhea with significant *p*-values (Table 2). Because the interference of other factors was not controlled for during the single-factor regression analysis, the results may not be reliable. Therefore, it is necessary to conduct further multiple-factor regression analyses. In this study, multiple-factor regression analysis using the maximum likelihood method revealed that the *p*-values of PAstV, PKoV, BVDV, and PEDV were lower than 0.05, and Exp (B) values were higher than 1, indicating that these five pathogens had a significant relationship with porcine diarrhea (Table 3). The regression equation was logit (P) = −46.439 + 0.734PAstV(1) + 1.554PKoV(1) + 18.787PoSaV(1) + 18.499PDCoV(1) + 1.747BVDV(1) + 3.709PEDV(1), with a prediction accuracy of 89.5%. Furthermore, statistical analysis showed that co-infection correlations existed among PKoV, PoSaV, PDCoV, BVDV, and PEDV (Figure 5, Table 4).

## 4. Discussion

Diarrheic diseases in swine have strong economic impacts on production units around the world. In recent years, porcine coronaviruses have represented some of the principal causes of these diseases [15]. PEDV has been described as the major pathogen that causes diarrheal outbreaks in pigs. However, reports of naturally occurring coinfection of coronaviruses with other viral agents highlight the importance of less-characterized viruses on disease severity and outcome [16]. It has been confirmed that PKoV, PSV, and PAstV are also responsible for gastrointestinal diseases, although they have been detected in fecal samples of animals that did not exhibit any clinical signs [17,18,19]. The close association between PKoV, PSV, and PAstV with neonatal piglet diarrhea has been previously reported [20,21,22] suggesting that these viruses may play synergistic roles in causing diarrheal disease. Wu et al. confirmed that PKoV enhanced PEDV pathogenicity and altered the number of intestinal lymphocytes in piglets [23]. Our group also identified that PSV and PEDV co-infections could aggravate the clinical symptoms observed in piglets (data not published). This could be attributed to an ineffective piglet immune response towards the viruses causing porcine diarrheal disease. At present, commercial vaccines comprise mainly inactivated or live viruses to induce an immune response against PEDV, TGEV, and PoRV, and these vaccines are not effective against the current swine epidemic pathogens. Findings from this study propose that the development of effective vaccines against a combination of PEDV and the novel co-infecting diarrheal pathogens (PSV, PKoV, PAstV, etc.) is an important direction to pursue.

This study demonstrates the dominant infection patterns of porcine diarrheal pathogens in Shanghai, China. The increasing epidemic trends of emerging diarrheal viruses are not limited to China but have also been reported in other countries, including Korea [24,25], Thailand [26], France [27], Japan [28], USA [29,30,31], Italy [32], Switzerland [18], India [33,34], and Croatia [35]. Therefore, it is imperative that greater attention is paid to these emerging diarrheal pathogens. PEDV can cause serious clinical symptoms, while most of the novel diarrheal pathogens also exist in healthy pigs albeit with higher rates detected in co-infection clinical samples. This suggests that these viruses may be synergistically interacting with PEDV, which makes clinical diarrhea prevention and control difficult.

The logistic regression model has been a routine technique for epidemic risk assessment. Though the emerging diarrheal pathogens were prevalent in pig herds with high detection rates, their risk of infection and epidemic is still unknown. In this study, two kinds of logistic regression models were compared. The single-factor logistic regression model showed that all 11 pathogens were closely related to porcine diarrhea, while the multiple-factor logistic regression model revealed that only PAstV, PKoV, BVDV, and PEDV were closely related to porcine diarrhea. Additionally, PEDV/PKoV, PEDV/PoSaV, PKoV/BVDV, PoSaV/BVDV, and PDCoV/PoSaV had great co-infection dominance. This may provide pathogen models for research on the co-infection pathogenesis.

In conclusion, the dominant co-infection models of porcine diarrheal pathogens were explored in this study and found that PEDV mainly mix-infected with the emerging diarrheal pathogens (PKoV, PAstV, and PSV). Further logistic regression analysis confirmed that emerging pathogens of PAstV and PKoV were significantly related to porcine diarrhea. An enhanced warning should be raised. However, additional techniques should be applied to conduct more comprehensive co-infection risk assessments.

## Figures and Tables

**Table 1:** Multiplex PCR primers.

Names	Gene	Primer Sequences	Length (bp)
PEDV-F	M	GATACTTTGGCCTCTTGTGT	332
PEDV-R		ATTGACTTACCTGTACGC	
TGEV-F	N	ACCAGATAGAAGTCACGTT	226
TGEV-R		TCAACCTGTGTGTCATCAAA	
PoRV-F	VP6	ATTTATATTYCATGCTAC	167
PoRV-R		CTGTCCAATTCATYCCT	
BVDV-F	N^pro^	TTACGACATCAACGGAT	372
BVDV-R		TGGTCCCTAGTCGCTCT	
PTV-F	3D	ATGGGACTCTAGATCTCGT	176
PTV-R		ACGCCTCTGTAGTTCTCTCTT	
PAstV-F	ORF2	GGATTTACAGTTGGCCCAGAT	249
PAstV-R		CCTGTCCATCTGCCTTTCTGT	
PKV-F	3D	ATGCTGCTTGGTGGACTCATT	280
PKV-R		ACGTCTCGTTGCCAAAGACAT	
PoSaV-F	ORF1	CGAGGCTAAAGGGAAAAACAAACGT	340
PoSaV-R		ACTGTCGTAGGTGTCTGTTTT	
PToV-F	S	CTTTTACACCTTGCCATCC	421
PToV-R		TGCTTCACCTCTACACTGTT	
PDCoV-F	M	ACTTATTCTGCTTTGGCTGCT	504
PDCoV-R		GAAGTGGTTATGGTGTGAAGTC	
PSV-F	5′UTR	GATGTGGCGCATGCTCTT	624
PSV-R		TGCTGCCTCCTGTGTTGTTAT	

**Table 2:** Variables not in the equation.

		Score	df	Sig.
Step 0 Variables	PTV	2.972	1	0.085
	PAstV	25.901	1	0.000
	PKoV	48.619	1	0.000
	PoSaV	14.489	1	0.000
	PToV	2.012	1	0.156
	PDCoV	6.892	1	0.009
	PSV	9.607	1	0.002
	BVDV	8.485	1	0.004
	TGEV	2.491	1	0.115
	PEDV	45.179	1	0.000
	PRoV	2.132	1	0.144
	Overall Statistics	125.131	11	0.000

**Table 3:** The variables in equation.

		B	S.E.	Wald	df	Sig.	Exp(B)
Step 1(a)	PAstV	0.734	0.231	10.129	1	0.001	2.083
	PKoV	1.554	0.286	29.625	1	0.000	4.730
	PoSaV	18.787	3503.178	0.000	1	0.996	1.443 × 10^8^
	PDCoV	18.499	4793.354	0.000	1	0.997	1.082 × 10^8^
	BVDV	1.747	0.724	5.820	1	0.016	5.739
	PEDV	3.709	1.006	13.603	1	0.000	40.806
	Constant	−46.439	5937.043	0.000	1	0.994	0.000

**Table 4:** *p*-values of pathogenic co-infection of swine diarrhea.

ID	PKoV	PoSaV	PDCoV	BVDV	PEDV
PKoV	0	0.92691	0.52696	0.00376	0.00276
PoSaV	0.92691	0	0.00097	0.00025	1.04 × 10^−6^
PDCoV	0.52696	0.00097	0	0.34198	0.91645
BVDV	0.00376	0.00025	0.34198	0	0.88815
PEDV	0.00276	1.04 × 10^−6^	0.91645	0.88815	0

## Data Availability

The original contributions presented in this study are included in the article. Further inquiries can be directed to the corresponding author.
